# Protein-constrained models pinpoints the role of underground metabolism in robustness of metabolic phenotypes

**DOI:** 10.1016/j.isci.2025.112126

**Published:** 2025-02-28

**Authors:** Maurício Alexander de Moura Ferreira, Eduardo Luís Menezes de Almeida, Wendel Batista da Silveira, Zoran Nikoloski

**Affiliations:** 1 Department of Microbiology, Federal University of Viçosa, Viçosa, Minas Gerais 36570900, Brazil; 2 Bioinformatics, Institute of Biochemistry and Biology, University of Potsdam, 14476 Potsdam, Germany; 3 Systems Biology and Mathematical Modelling, Max Planck Institute of Molecular Plant Physiology, 14476 Potsdam, Germany

**Keywords:** Systems biology, In silico biology

## Abstract

Integrating enzyme parameters into constraint-based models have significantly improved the prediction of physiological and molecular traits. To further improve these models, we integrated promiscuous enzyme activities that jointly comprise the so-called underground metabolism by developing the CORAL toolbox, which increases the resolution of modeled enzyme resource allocation. Applying CORAL to a protein-constrained model of *Escherichia coli* revealed that underground metabolism resulted in larger flexibility of metabolic fluxes and enzyme usage. Simulating metabolic defects where the main activity of a promiscuous enzyme was blocked but promiscuous activities remained functional showed a small enzyme redistribution to the side activities. Further, blocking pairs of main activities showed that non-promiscuous enzymes exhibited larger impact on growth than promiscuous enzymes. These simulations showed that promiscuous enzymes can compensate for these defects, in line with experimental evidence. Together, our results indicated that promiscuous enzyme activities are vital to maintain robust metabolic function and growth.

## Highlights

• CORAL toolbox integrates promiscuous enzyme activity into enzyme-constrained models
• Inclusion of these activities increases metabolic flux variability
• These activities improve metabolic flexibility in simulations of metabolic defects
• Enzymes with promiscuous activities are important for cell survival and growth

## Graphical abstract

**Figure:**
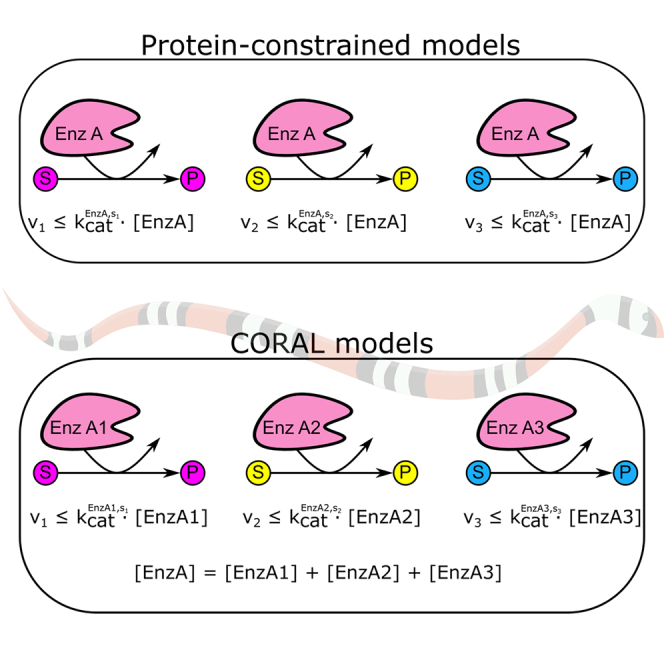

## Introduction

Enzymes are the workhorses of metabolism. They catalyze the conversion of substrates into products for the majority of metabolic reactions. Although some enzymes are highly specific with respect to reactions they catalyze, there is notable enzyme promiscuity, with estimates of some enzymes catalyzing reactions with hundreds of different metabolites serving as substrates.^1^ Promiscuous enzymes catalyze more than one reaction by binding with smaller affinity to other substrates.^2^ As a consequence of lower substrate affinity, the side reactions catalyzed by promiscuous enzymes occur at a lower rate than the main reaction. Promiscuous enzymes also exhibit lower catalytic efficiency for the side reaction in comparison to the main reaction.^3^ As a result, it has been taught that side reactions are physiologically irrelevant.^4^ However, these side reactions still happen often enough to form an alternative metabolic network, termed underground metabolism.^5^ More precisely, underground metabolism is defined as the metabolic network of reactions catalyzed by enzymes acting on substrates that are not their native substrates.^5^ These alternative substrates are endogenous to the cell and have lower affinity to the enzymes in the promiscuous reactions.^3^ This underground metabolic network serves an important role in evolution, providing a reservoir of enzyme functions to evolve via natural selection.^6^ Evidence indicates that gene duplication events contribute to underground metabolism by creating copies of the enzyme, with one copy evolving higher affinity to the substrate of a certain side reaction.^7^ Further, underground metabolism can also be used for biotechnological purposes, aiding adaptive laboratory evolution (ALE) experiments,^8^ and guiding metabolic engineering efforts.^9^

Constraint-based approaches rely on optimization principles to predict and study metabolic phenotypes using genome-scale metabolic models (GEMs).^10^ GEM-based investigations of underground metabolism have provided useful insights, such as the connectivity between native and underground metabolism and how underground reactions contributes to adaptation to new environments,^6^ improved gap-filling of GEMs,^11^ and the design of metabolic engineering strategies.^9^ While insightful, these investigations were performed using conventional GEMs, which do not consider constraints on the available enzyme abundance and enzyme catalytic rates. Consideration of these constraints has resulted in the generation of protein-constrained GEMs (pcGEMs) that have been shown to improve predictive performance.^12^^,^^13^^,^^14^

The GECKO toolbox^13^^,^^15^^,^^16^ allows for a straightforward reconstruction of pcGEMs, leveraging kinetic data from available databases and integrating data-driven predictions of enzyme catalytic rates for most enzymes in a model. The GECKO formulation of pcGEMs also allows for predicting how much resource is allocated to a particular enzyme. The amount of resources that each enzyme can have is constrained by the total amount of experimentally measured total protein content. The GECKO formulation assumes, however, that an enzyme that catalyses multiple reactions has the same amount of resources (i.e., abundance of enzymes) for all the reactions it catalyses. In a biological context, enzymes are almost always occupied by their main substrates,^17^ such that enzyme resources are allocated mostly to the main reactions. This means that the availability of enzyme resources for side reactions is drastically reduced. This issue is not accounted in the existing pcGEM approaches. Further, it remains elusive how the metabolic network allocates enzyme resources between main and side reactions.

To address these questions, here we propose an approach for modeling promiscuous enzyme activity in pcGEMs, termed *co*nstraint-based p*r*omiscuous enzyme and underground met*a*bolism mode*l*ing (CORAL) toolbox. The CORAL toolbox is developed for MATLAB and builds on GECKO 3 to predict enzyme allocation while ensuring that the main and promiscuous activities have separate resource pools. We show that CORAL can predict the distribution of resources among main and side reactions, making it a useful approach to understand promiscuous enzyme activity and underground metabolism. In addition, we show how CORAL can be used to gain insights in the functional implications of promiscuous activities in metabolic networks.

## Results and discussion

### CORAL accounts for promiscuous enzyme activity and underground metabolism

By building upon existing protein-constrained approaches, we developed CORAL as a toolbox to investigate promiscuous enzyme activity and underground metabolism in the context of constraint-based modeling. To this end, we first included underground reactions into the *E. coli* iML1515 model, resulting in the iML1515u model. Inspection of the model identified no duplicated or highly similar reactions resulting from the addition of underground reactions. Then, using the DLKcat-$k_{cat}$ values, we used GECKO 3 to integrate enzyme constraints into iML1515u. The pcGEM was then restructured using CORAL, and the resulting model was named eciML1515u.

**Figure 1:**
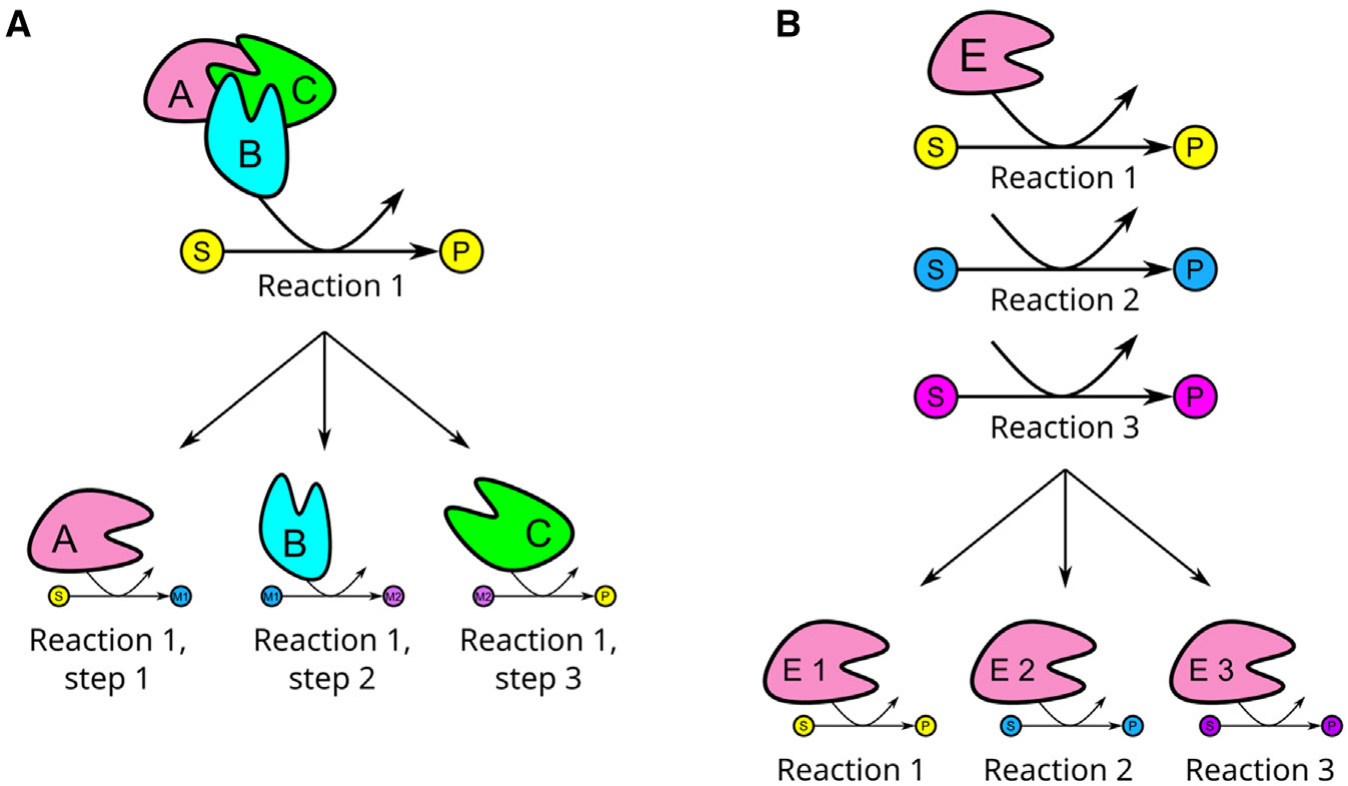
Restructuring of the pcGEM as performed in CORAL (A) Simplification of GPR rules to split reactions catalyzed by enzyme complexes into multiple partial reactions. (B) Splitting of promiscuous enzymes into subpools of enzymes, each catalyzing a single reaction.

In CORAL, the enzyme usage in reactions is restructured in a manner that allows for modeling resource usage in promiscuous enzymes. This is achieved by splitting the enzyme pool for each promiscuous enzyme into as many subpools as there are side reactions (Figure 1). The sum of subpools for a certain enzyme then corresponds to the original enzyme pool (see Equations 5, 12, and 19, STAR Methods). Before the restructuring, the model contained 3774 metabolites, 8331 reactions, and 1526 enzymes, and application of CORAL resulted in eciML1515u that contains 12048 metabolites, 16605 reactions, 1526 enzymes, and 7260 subpools (Table 1). The large number of metabolites and reactions is due to the added pseudometabolites and pseudoreactions to accommodate the subpools and their usage of the enzyme pools, along with simplification of GPR rules to split enzyme complexes into partial reactions.

**Table 1:** General descriptors for the models used in this study

	iML1515	iML1515u	eciML1515u (GECKO)	eciML1515u (CORAL)
Genes	1516	1530	1531	1531
Reactions	2712	3238	8331	16605
Metabolites	1877	2247	3774	12048
Enzymes	–	–	1526	1526
Enzyme subpools	–	–	–	7260

### Promiscuous enzymes increase metabolic flux variability

**Figure 2:**
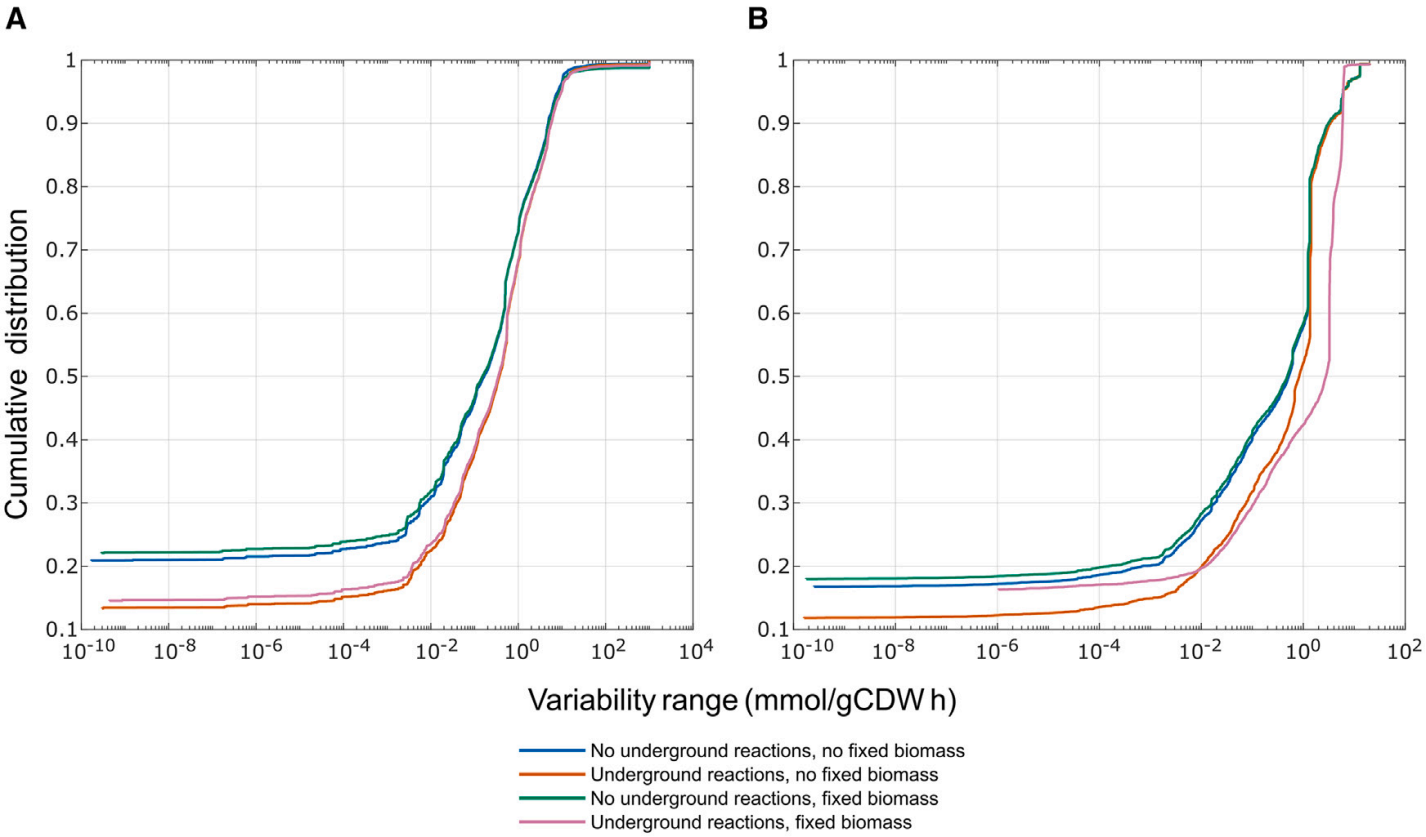
Flux variability for the CORAL-restructured model We investigated flux and resource allocation variability with or without underground reactions and with or without fixed biomass, as indicated in the legend. (A) Simplification of GPR rules to split reactions catalyzed by enzyme complexes into multiple partial reactions. (B) Splitting of promiscuous enzymes into subpools of enzymes, each catalyzing a single reaction. See Figure S4 for TurNuP results.

Promiscuous enzyme activity and underground metabolism provide alternative flux routes. To investigate the impact of these added reactions, we performed flux variability analysis (FVA) using eciML1515u, both with and without underground reactions. We further tested how chemostat conditions affect flux variability by using a fixed growth rate. We found that flux variability is higher when underground reactions are present (Figure 2A). The scenario without underground reactions and no fixed growth rate had a lower flux variability in 79.85% of reactions when compared to the condition with underground reactions and no fixed growth rate. The scenario with fixed growth rate and without considering underground reactions showed lower flux variability in 79.22% of reactions in comparison to the condition with underground reactions and fixed growth rate.

Next, we performed FVA to check the variability of subpool usage instead of metabolic flux. We found that subpool usage variability behaves similarly to metabolic flux variability, with underground reactions leading to an increase in the overall variability (Figure 2B). The subpool usage range was larger in 82.13% of subpools for the condition with underground reactions and no fixed growth rate. Similarly, the subpool usage was larger in 83.30% of subpools for the condition with underground reactions and a fixed growth rate. Therefore, we concluded, in line with the expectation, that underground metabolism leads to higher variability of metabolic fluxes and enzyme usage.

### Promiscuous enzyme activities ensure metabolic robustness by redistribution of enzyme resources from main to side reactions in response to metabolic defects

The restructuring of enzyme usage in CORAL allows for a closer inspection of resource allocation, as the enzyme usage pool for promiscuous enzymes are now split into different subpools for each reaction it catalyses. Given that, biologically, the larger part of enzyme resources is allocated to the main reaction,^17^ we investigated how these resources are distributed to the side reactions when metabolic defects prevent the main reaction from occurring. To this end, we performed a simulation where we blocked the main reaction by setting its enzyme subpool to zero, ensuring that there is no enzyme subpool available to catalyze the main reaction. This is necessary as performing gene knockouts using conventional GPR rules would impact all subpools of a certain enzyme, along with disturbing additional reactions. Further, doing this would prevent the promiscuous reactions to step up and compensate for the loss of the main function of the enzyme, which is not biologically realistic and is evidenced to occur in experimental analyses.^7^

In a first round of simulations, we determine the enzyme subpool usage distribution in the wild type by solving an optimization problem (P3, STAR Methods). Next, we used the solution of this problem to constrain the enzyme usage distributions in the disturbed network that abolishes the main activity. These simulations were performed under the same constraints on substrate uptake rates to favor a respiratory metabolism. We found a total of 30 optimal solutions where the main reaction enzyme subpool was predicted to be used in the wildtype simulation and that resulted in non-lethal defects after being blocked in the second simulation.

Considering the changes occurring within an enzyme pool, 7 of the 30 solutions resulted in an almost direct redistribution of $E_{s,1}$ to $E_{s,2}$ (which has the second highest catalytic efficiency), such that the enzyme subpool usage value for $E_{s,2}$ in the defective network solution is the same as for $E_{s,1}$ in the WT solution or within 5% proximity (since we allow for 5% flexibility). For instance, this was the case for the tryptophanase TnaA, whose main subpool is used in the reaction where the enzyme catalyses the degradation of tryptophan, but the $E_{s,2}$ subpool is used in the reaction where the enzyme catalyses the degradation of serine. For 13 other solutions, including: TyrB and ArgA, there is a higher allocation to $E_{s,2}$, while other subpools also receive resources but at a lesser amount. The main subpool of these enzymes are used in the reaction where the enzyme catalyses reactions in the biosynthesis of amino acids, as well as the other subpools, although for different amino acids. For seven solutions, including ProC and MtnN, other enzyme subpools received more resources than $E_{s,2}$. Both are also involved in amino acid biosynthesis, also having side subpools used in reactions where the enzyme is catalyzing reactions for different amino acids other than the one in the main reactions. Lastly, in ten solutions, the subpool $E_{s,1}$ is redistributed entirely to enzyme subpools other than $E_{s,2}$ (see Table S2). For instance, this was the case for TmkK, whose main subpool is used in the reaction where the enzyme phosphorylates dTMP to dTDP, and whose other subpools is used in the reaction where the enzyme phosphorylates other dNMP nucleotides into dNDPs.

To assess the magnitude of the changes in enzyme subpool allocation after blocking the main reaction, we calculated the ratio of how much resource each enzyme subpool draws from the enzyme pool ($E_{s,j}/E_{s}$). We found that none of the enzyme subpools that showed an increase in the defective network compared to the wildtype were used in the wildtype solution. Inspecting the enzyme subpools with the highest ratio after the main reaction being blocked, the one with the highest increase belongs to the enzyme pool of the outer membrane porin C, which has 566 subpools. Its main enzyme subpool is used in the reaction where the enzyme catalyses the transport of calcium into the cytosol. Before the main reaction being blocked, the main enzyme subpool takes 98.03% of the enzyme pool, whereas after the main reaction is blocked, the resources were used instead by the subpool 522, which takes up 98.12% of this enzyme pool. The enzyme subpool 522 is used in the reaction where the enzyme catalyses the transport of L-tryptophan to outside the cell. Another subpool that takes over the enzyme pool is the subpool 2 of the amino acid acetyltransferase. The subpool 2 is used in the reaction where the enzyme catalyses the transfer of the acetyl group from acetyl-CoA into DL-2-aminopimelate, forming 2-acetylaminoheptanedioate and coenzyme A, taking up 83% of the enzyme pool.

**Figure 3:**
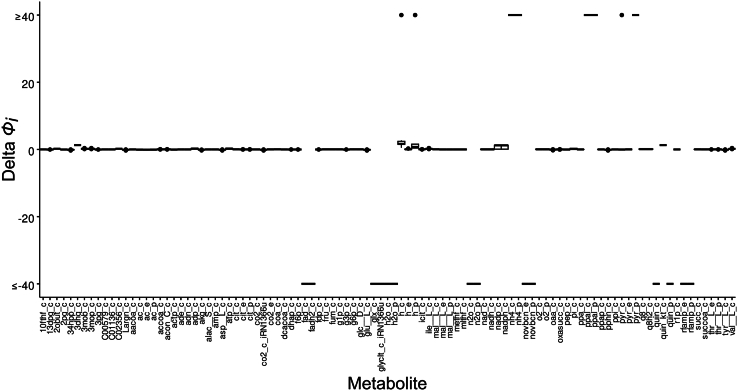
Difference in flux-sums between the wildtype and the defective network solutions We calculated the flux sums only for metabolites that were common to all solutions when blocking a single main reaction. We calculated the values by subtracting the flux-sums of both solutions ($\phi_{i}^{del}-\phi_{i}^{WT}$). Only a few metabolites were impacted, namely NAD+/NADH, NADP+/NADPH and UDP-N-acetyl-D-glucosamine (see Section 3.1). The low impact of these metabolic defects suggests that alternative pathways can use these substrates and compensate for the loss of function. Metabolites with flux-sums of 1000 or −1000 were rescaled to allow better visualization of smaller values. Full names of the metabolites appearing on the x axis are provided in Table S4. See Figure S4 for TurNuP results.

Considering the global changes in the model following the blocks in the main reaction, we calculated the difference between enzyme subpool usage predicted for the wild type and for all the defective network simulations. In most solutions, the enzyme subpool usage, with few exceptions, shows a slight change (Figure S1). Among the enzyme subpools with the largest change, there are the enzyme subpools 2 and 3 of the subunit alpha of the ATP synthase complex. Another affected enzyme pool is the subunit beta of the ATP synthase complex, of which the enzyme subpools 2 and 3 also have a higher increase compared to subpools in other enzyme pools. The enzyme subpools 2 and 3 of the ATP synthase gamma chain are also highly impacted. These enzyme subpools are allocated the largest part of the enzyme pool after the blocking of several different main reactions. These enzyme subpools are used in the reaction where the enzyme catalyses the inverse reaction of ATP synthase, producing ADP from ATP hydrolysis. It is important to highlight that these metabolic defects were not introduced *in tandem*, i.e., subunits alpha, beta and gamma are blocked together, but instead that each subunit of ATP synthase was blocked individually in separated simulations, which could be a reason why ATP and ADP flux-sums are not impacted (Figure 3). Nonetheless, this result shows that when the main reaction of ATP synthase is blocked, it instead redirects flux to ATP hydrolysis. In *E. coli*, the synthesis or hydrolysis of ATP depends on the direction of mechanical rotation of the ATP synthase complex. This means that CORAL can accurately predict changes in enzyme resource usage for reversible reactions where the reverse reaction has a lower catalytic efficiency.

Given that in this analysis the main reaction is blocked, we inspected if the metabolites involved in these reactions display any change in flux-sums, which can be a useful proxy for metabolite concentrations.^18^^,^^19^ We calculated the flux-sums of the wild type solution and the solutions of all 38 defective network simulations and calculated their differences (see STAR Methods). Surprisingly, we found that very few metabolites display changes in flux-sums (Figure 3). Notably, both NAD+/NADH and NADP+/NADPH presented slightly reduced flux-sums, suggesting that in some solutions, there is a disturbance of redox balance. This can have an impact on central metabolic pathways, since these cofactors are the key electron donors in the respiratory chain and in anabolic reactions. This also leads to oxidative stress and impaired growth and cell survival.^20^^,^^21^ In our simulations, however, we do not observe a decrease in growth rate. The metabolite with increased flux-sum is UDP-*N*-acetyl-D-glucosamine, which is the precursor of peptidoglycan and is involved in the biosynthesis of lipopolysaccharides.^22^ Taken together, these results indicate that while a few metabolites are impacted in certain solutions, the cell metabolism is robust to disturbances in enzymes with promiscuous activity, suggesting that having alternative routes can mitigate the impacts of metabolic defects.

This simulation setup highlights the advantage of CORAL in comparison to other pcGEM formulations, such that it makes it possible to directly assess the importance of promiscuous enzymes and provides a more biologically realistic formulation to integrate enzymes in a GEM. Further, it is biologically realistic in comparison to similar experimental setups. Patrick et al.^23^ evaluated how new metabolic functions can originated in *E. coli* by performing a combination of gene knock outs and gene overexpressions. They performed a mutant assay where 104 genes were knocked out, resulting in auxotrophic strains, and then overexpressed several unrelated genes. They found that as many as 20% of auxotrophic mutants could be “rescued” by these genes being overexpressed. This experimental setup matches our simulation setup in the sense that we also cause metabolic defects that are also “rescued” by promiscuous activities. Another experimental work, performed by Jöst et al.,^24^ focused on covalent inhibitors and their promiscuous activity across different enzymes. They have shown that even when selective inhibition is attempted, some enzymes can still react with different substrates. This indicates that blocking an enzyme’s main activity while allowing its promiscuous activities is experimentally feasible. Although a high-throughput, genome-scale assessment could be a challenging endeavor, especially for eukaryotes, where gene knock ins and knock outs are less trivial than in prokaryotes,^25^ many genes can be tested individually. One could engineer a promiscuous enzyme to have a lower affinity to its main substrate, blocking or severely reducing the main reaction while allowing the modified enzyme to catalyze its side reactions. There have been many advances in enzyme design that could allow for such changes in the substrate specificity.^26^^,^^27^^,^^28^ Specifically, the substrate multiplexed screening^29^ (SUMS) approach has been used for engineering the substrate specificity of *Ruminococcus gnavus* tryptophan decarboxylase (RgnTDC). In the study, it was observed that many mutations appeared to have neutral or negative effects on the engineered enzyme’s capability of catalyzing certain substrates but had positive effects for other substrates. We suggest that similar analyses could be useful for assessing the impacts on blocking main reactions. CORAL thus makes it possible to test the impact of these metabolic defects in a much larger scale than currently possible either *in vivo* or *in vitro*, proving to be a useful tool for generating hypotheses and guiding experimental work.

### Impacts of metabolic defects on growth are mitigated by enzymes with promiscuous activity

We next assessed how growth is impacted when pairs of main reactions are blocked. To this end, we blocked a pairwise combination of all main reactions by setting the subpool of each reaction (e.g., $E_{1,1}$ and $E_{2,1}$) to zero and compared how it changes compared to a growth rate of 0.1 h^−1^ for the wild type (WT). Altogether, we obtained 3309 pairs of enzymes whose main reaction subpool blockage reduced this growth rate by least 1%. The enzyme subpool pairs used a combination of 156 different enzymes, of which 68 enzymes have promiscuous activity in the model.

Inspecting the blocked pairs that most impacted growth (higher than 1% of the growth rate), two enzymes stand out as causing the growth rate to drop no matter the other component of the pair, with the 618 most impacted pairs out of 3309 containing either of these two enzymes. These are the inosine-5′-monophosphate dehydrogenase, whose main activity in the model is the conversion of inosine-5′-phosphate (IMP) to xanthosine-5′-phosphate (XMP); and the probable acyl-CoA dehydrogenase YdiO, whose main reaction in the model is the conversion of crotonoyl-CoA into butanoyl-CoA using one reduced FAD. Further, blocking both of these enzymes leads to the largest reduction in the growth rate, with a ratio of growth to that of the wild-type of 0.57. The metabolite xanthosine-5′-phosphate is an intermediary of the *de novo* synthesis of guanine nucleotides, thus being important for growth. Likewise, butanoyl-CoA is an intermediate metabolite in fatty acid metabolism, in both β-oxidation and elongation in mitochondria. In the CORAL model, these enzymes have no promiscuous activity, meaning they have no enzyme subpools that could be used in different than the main reaction, such that no compensatory effect is possible.

**Figure 4:**
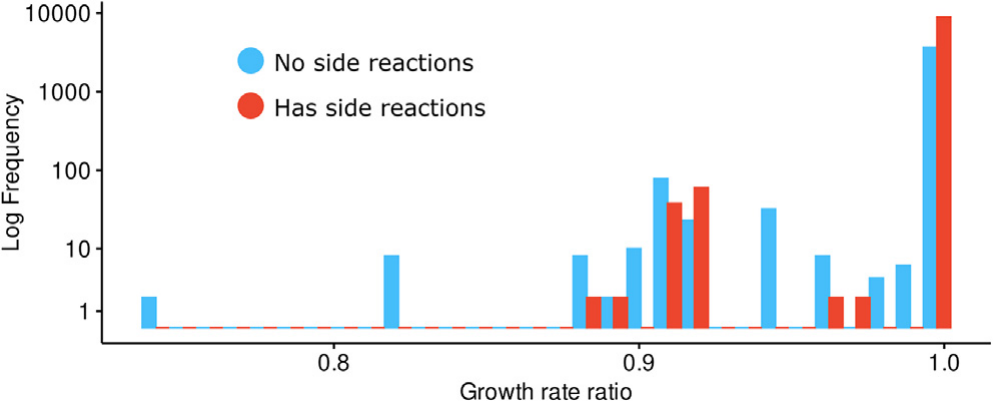
Distribution of growth rate ratios (μWTμdel) when blocking pairs of main reactions Growth rate ratios were calculated for all main reaction pairs, comparing these to the WT solution. The distribution of growth rate ratios after blocking pairs of reactions shows that the pairs resulting with the largest impact on growth were pairs with no promiscuous activity. Given that most pairs with no effect on growth were promiscuous pairs, this suggests that alternative pathways can compensate for the loss of function to maintain the growth rate. See Figure S4 for TurNuP results. See Table S3 for the list of subpool pairs for blocked pairs of main reactions.

Among the 20912 enzyme pairs that caused no impact on growth (equal or less than 1% of the growth rate), in 15052 pairs there is at least one enzyme with promiscuous activity (Figure 4), indicating that these have resources redistributed to other enzyme subpools upon blocking the main reaction enzyme subpool. Meanwhile, in the pairs that affect growth, there is a higher prevalence of enzymes without promiscuous activity (Figure 4; Table S3).

**Figure 5:**
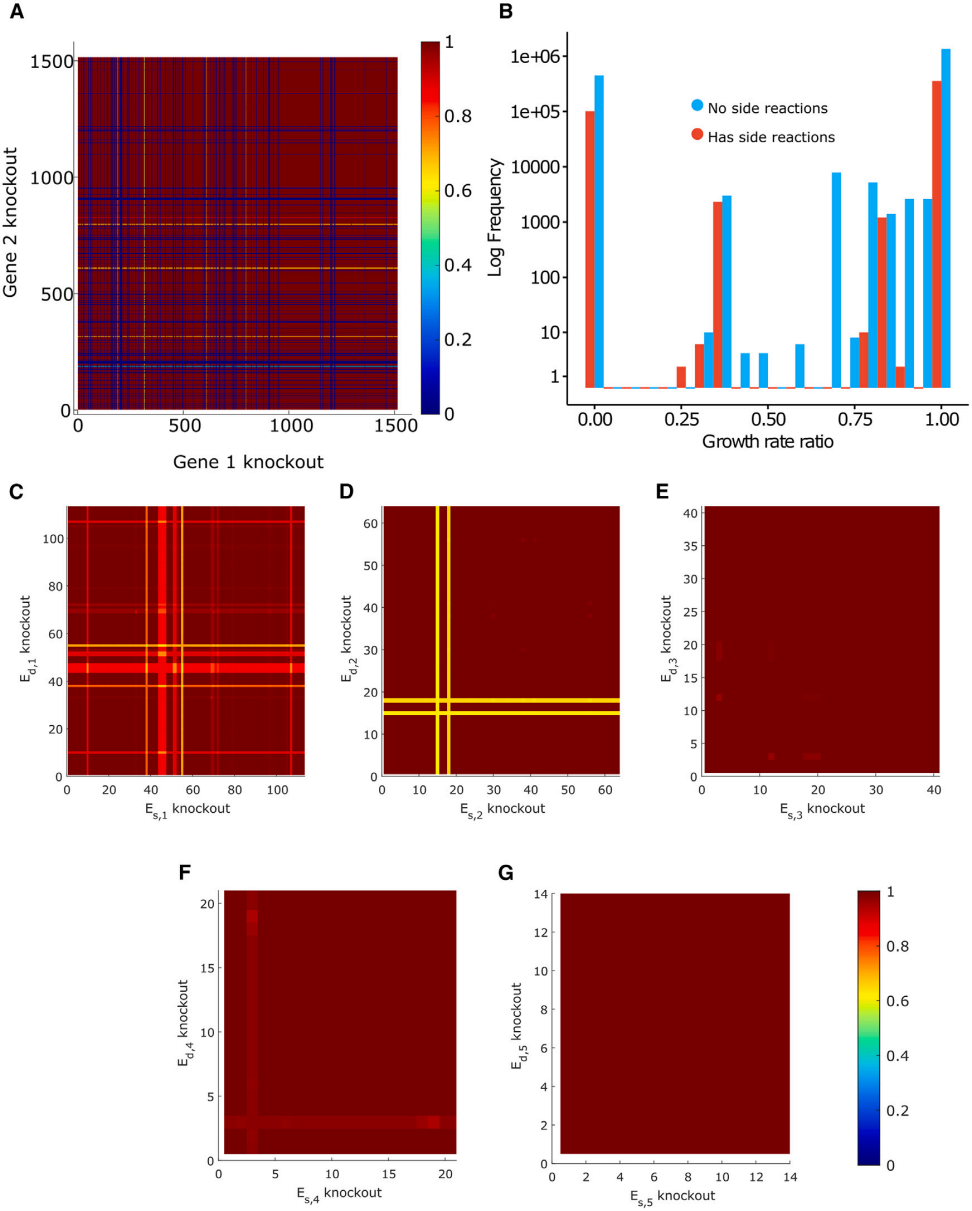
Impact on growth after blocking pairs of main reactions (A) Impact on growth following double gene knockouts in the iML1515u model, performed by deleting pairs of genes according to GPR rules. (B) Distribution of growth rate ratios following double gene knockouts in the iML1515u model. (C) Impact on growth after blocking a pair of main reactions (first subpool, $E_{s,1}$) in the CORAL model. (D) Second subpool ($E_{s,2}$). (E) Third subpool ($E_{s,3}$). (F) Fourth subpool ($E_{s,4}$). (G) Fifth subpool ($E_{s,5}$). See Figures S2 and S3 for results using other carbon sources and Figure S4 for TurNuP results.

To further assess the effects of the double metabolic defects on growth, we also knocked out pairs of genes from the conventional GEM iML1515u (Figure 5A). We knocked out pairs of genes following GPR rules for all reactions. This is useful to assess how much enzyme promiscuity matters for maintaining the growth rate, since now all reactions controlled by the knocked-out gene will no longer occur. In this scenario, we find that this indeed results in lower growth rates for pairs of genes that code for promiscuous enzymes when no side reactions are available (Figure 5B), suggesting that when all reactions, main or side, catalyzed by an enzyme are blocked at the same time, the compensating effect observed in the CORAL model is lost (Figure 5C). Altogether, these results corroborate the findings that promiscuous reactions are important for the maintenance of the growth rate as well as mitigating impacts of defects on cell metabolism.

We also inspected what happens when we block any of the side reactions by setting the enzyme subpools from $E_{s,2}$ to $E_{s,5}$ to zero. We noticed that there is little impact on the growth rate. When blocking the reaction where the enzyme subpool $E_{s,2}$ is used (Figure 5D), there were four enzymes whose presence in a pair had the most impact had the growth rate ratio (≤0.71). For the enzyme subpools $E_{s,3}$ to $E_{s,5}$, the lowest growth rate ratio was 0.905 (Figures 5E–5G). In contrast, blocking pairs of main reactions had larger impact on the growth rate. This suggests that while side reactions are essential to maintain metabolic and growth balance when the main reaction is impacted, in the opposite situation—blocking side reactions while enabling the main reaction—the double metabolic defects have negligible impact on growth. This observation holds when growth on different carbon sources are evaluated (Figure S2), even when using organic acids as carbon source instead of sugars (Figure S3).

### Predictions from CORAL are robust to $k_{cat}$ values obtained from another predictive tool

The GECKO 3 version has the DLKcat tool integrated into its pcGEM reconstruction pipeline. This has expanded the capabilities of pcGEM reconstruction, given that databases such as BRENDA often have inaccurate or incomplete information, especially for non-model species.^30^ However, DLKcat has been subject to scrutiny given that its predictions of $k_{cat}$ values are poor for enzymes that differ too much from those in the training dataset.^31^ To assess the robustness of our results, we parameterized the underground reactions of eciML1515u with $k_{cat}$ values predicted using TurNuP^32^ and repeated all analyses. We focused on underground reactions since these have non-typical enzyme/substrate pairs and provide a reasonable challenge to $k_{cat}$ prediction tools.

First, we compare the $k_{cat}$ values obtained from DLKcat and TurNuP for underground reactions. Interestingly, we found a poor correlation between the two $k_{cat}$ sets, with a Pearson correlation of 0.21 (Figure S4A). Regarding flux and enzyme usage variability, our results indicated that that the addition of underground reactions still increases the variability in flux and enzyme usage (Figures S4B–S4C). Regarding single metabolic defects, the same metabolic robustness was observed when investigating flux-sums, having most of the same metabolites being impacted (Figure S4D). For the double metabolic defects that impact growth, we found that the same enzyme subpools are responsible for the decrease in the growth rates as in the DLKcat predicted $k_{cat}$ values (Figure S4E). Lastly, we also observed that in the pairs that affect growth, there is a higher prevalence of enzymes without promiscuous activity (Figure S4F). Taken together, these results show that the predictions obtained with CORAL are robust to the choice of $k_{cat}$ prediction tool. Given the poor correlation between the two sets of $k_{cat}$ values, this might be attributed to a compensatory effect by enzyme abundances. Further, considering that native metabolism is governed by reactions catalyzed by the main enzyme subpool, disturbances in the side reactions could have limited impact on cell physiology, especially growth.

### Conclusion

Here, we present the CORAL toolbox, a tool for the integration and analysis of promiscuous enzyme activity and underground metabolism. We demonstrated that the inclusion of underground reactions increases metabolic flux variability. Furthermore, the redistribution of enzyme resources from the main to the side reactions highlights the importance of underground metabolism to maintain metabolic flexibility and robustness, serving as alternative routes that compensates for the loss of the main reaction. Moreover, when inspecting the impact of double metabolic defects on growth, promiscuous enzyme activity and underground metabolism proved essential to maintaining growth, as the highest impact were on enzymes without promiscuity. Further, when simulating a scenario where promiscuous enzyme activity does not take place, we observe loss of this compensating effect. Lastly, these results are robust to $k_{cat}$ values obtained from a different predictive tool. These results highlight the importance of considering underground metabolism and enzyme promiscuity in terms of metabolic plasticity in constraint-based approaches. The introduced approach allows comprehensive investigations of the interaction between underground metabolism and the rest of the network as well as adaptation to disturbances, pinpointing that flexibility is crucial for cell survival and resilience.

### Limitations of the study

We note that all of the findings are obtained under the constraint that the ratio of the enzyme allocation to a side reaction in comparison to the corresponding main reaction is reciprocal to the ratio of the respective turnover numbers. This assumption is equivalent to imposing equality between the maximal velocities of the main and side reactions. While, in absence of extensive data on maximal velocities for promiscuous enzymes, this assumption may be plausible, other scenarios can be considered, such as imposing ordering of maximal velocities corresponding to the ordering of turnover numbers. We expect that this relaxation can lead to additional insights about functional implications of promiscuous enzymes.

## Resource availability

### Lead contact

Further information and requests for resources should be directed to and will be fulfilled by the lead contact, Zoran Nikoloski (zoran.nikoloski@uni-potsdam.de).

### Materials availability

This study did not generate new unique reagents.

### Data and code availability

• The CORAL Toolbox is publicly available in a GitHub repository along with the code and data for reproducing this work: https://github.com/mauricioamf/CORAL.
• Any additional information required to reanalyze the data reported in this paper is available from the lead contact upon request.

## Acknowledgments

We thank Dr. Marius Arend, Dr. Philipp Wendering, and Fayaz Soleymani for their support on this study. This study was financed in part by the Coordenação de Aperfeiçoamento de Pessoal de Nível Superior—Brasil (10.13039/501100002322CAPES)—Finance Code 001.

## Author contributions

M.A.d.M.F.: conceptualization, methodology, software, validation, formal analysis, investigation, writing—original draft, writing—review and editing. E.L.M.d.A.: conceptualization, methodology, writing—review and editing. W.B.d.S.: conceptualization, writing—review and editing, supervision, project administration, funding acquisition. Z.N.: conceptualization, methodology, writing—original draft, writing—review and editing, supervision, project administration, funding acquisition.

## Declaration of interests

The authors have declared no competing interests.

## STAR★Methods

### Key resources table

**Table:** 

REAGENT or RESOURCE	SOURCE	IDENTIFIER
**Deposited data**		

Underground iJO1366	Kovács et al.^9^	https://github.com/pappb/Kovacs-et-al-Underground-metabolism
iML1515	Monk et al.^33^	http://bigg.ucsd.edu/models/iML1515
*E. coli* maximum growth rate	Schmidt et al.^34^	https://doi.org/10.1038/nbt.3418
*E. coli* total protein content	Valgepea et al.^35^	https://doi.org/10.1039/C3MB70119K

**Software and algorithms**		

MATLAB	The MathWorks Inc., Natick, Massachusetts	https://www.mathworks.com/products/matlab.html
COBRA Toolbox 3	Heirendt et al.^36^	https://opencobra.github.io/cobratoolbox/stable/index.html
RAVEN Toolbox 2	Wang et al.^37^	https://github.com/SysBioChalmers/RAVEN
GECKO 3	Chen et al.^16^	https://github.com/SysBioChalmers/GECKO
DLKcat	Li et al.^38^	https://github.com/SysBioChalmers/DLKcat
TurNuP	Kroll et al.^32^	https://github.com/AlexanderKroll/kcat_prediction_function
CORAL Toolbox	This paper	https://github.com/mauricioamf/CORAL

### Method details

#### Refining and reconstruction of the models

To reconstruct a GEM accounting for underground metabolism, we manually curated the *Escherichia coli* GEM iML1515^33^ to include underground reactions . We included the reactions from the underground iJO1366^39^ model created in the Kóvacs et al. (2022) study.^9^ These were predicted by the PROPER algorithm, with 20% of these reactions validated experimentally.^6^^,^^40^ We integrated these reactions, matched all annotations from iJO1366 to the format used by iML1515, and standardized the gene-protein-reaction (GPR) rules, resulting in the iML1515u model. The list of added reactions is available in Table S1. Considering that iML1515 already includes many promiscuous reactions, we double-checked whether the inclusion of underground reactions from underground iJO1366 could have resulted in duplicate or highly similar reactions. To accomplish this, we checked in the stoichiometric matrix of iML1515u for reactions that are identical or have highly similar stoichiometric coefficients given cosine similarity with a 90% similarity threshold, which would be removed from the model if detected.

Next, we reconstructed the protein-constrained version of iML1515u using GECKO 3.^16^ We populated the adapter file with *E. coli*-specific information, using a maximum growth rate when not constrained by nutrient uptake of 0.6 h^−1^, ^34^ total protein content ($P_{tot}$) of 0.61 g_protein_/g_DW_,^35^ and the default value of 0.5 for average enzyme saturation factor (σ) and fraction of enzymes in the model (f). We followed the steps described to reconstruct the full ecModel, with constraints on individual enzymes. For all reactions in the model, we integrated $k_{cat}$ values predicted by DLKcat.^38^ We also built a model with $k_{cat}$ values predicted by TurNuP^32^ for underground reactions to assess how a different $k_{cat}$ prediction tool would affect the results. We followed the protocol until Stage 2 since Stage 3 deal with tuning model parameters as we wanted to preserve the original DLKcat-predicted $k_{cat}$ values. All model refinements were performed using the COBRA Toolbox 3^36^ and the RAVEN Toolbox 2^37^ in MATLAB (The MathWorks Inc., Natick, Massachusetts).

#### Restructuring the pcGEM

To account for the enzyme usage for individual reactions catalysed by a promiscuous enzyme, we developed the CORAL Toolbox, an extension of GECKO 3, and available for MATLAB. CORAL modifies the GECKO formulation and how enzymes are integrated in the model. The GECKO^13^^,^^15^^,^^16^ formulation of pcGEMs directly represents enzymes in the stoichiometric matrix, where each enzyme corresponds to a pseudometabolite that participates in a reaction, with a pseudo-stoichiometric coefficient given by the ratio between the molecular weight of the enzyme (MW) and its turnover number, $k_{cat}$, for the reaction (MW/ $k_{cat}$). The enzyme usage distribution is bounded by the total protein pool, which is represented as an exchange pseudoreaction, itself bounded by experimental data on total cell protein content. For each enzyme, there is a pseudoreaction assigning a share of the total protein pool to the enzyme. In this formulation, an enzyme that catalyses two or more reactions uses the same enzyme pseudometabolite in each reaction, indicating that the same amount of enzyme is used for all reactions catalysed by the enzyme. Given that enzyme abundance can be calculated by knowing the flux of a reaction and the catalytic rate of the catalysing enzyme, we split the pool of an enzyme that catalyses more than one reaction into multiple subpools. Then, one subpool is assigned for each promiscuous reaction catalysed by that enzyme, such that each subpool represents the enzyme resources of that single reaction only (Figure 1).

The restructuring of a GECKO 3-constructed pcGEM is done in three steps: (i) simplifying GPR rules; (ii) splitting enzyme pools into subpools for all reactions; and (iii) updating enzyme information on the model. The first step of the restructuring entails the simplification of the GPR rules. In GECKO 3, reactions catalysed by isozymes (“OR” rules) are separated into different reactions catalysed by a single enzyme. The CORAL Toolbox introduces another simplification, dealing with reactions catalysed by multiple enzymes (“AND” rules), by splitting all reactions catalysed by enzyme complexes into multiple partial reactions catalysed by one enzyme each. In the second step, we ordered the reactions catalysed by promiscuous enzymes from the lowest to the highest ratio of molecular weight (MW) to turnover number, i.e. MW/ $k_{cat}$. We defined as the main reaction the one with the largest $k_{cat}$ value (i.e., lowest MW/ $k_{cat}$ ratio); the other reactions catalysed by the same enzyme are considered side reactions. For each reaction catalysed by a promiscuous enzyme, a pseudoenzyme was created to replace the promiscuous enzyme. A number was then appended to the IDs of each pseudoenzyme according to the order of MW/ $k_{cat}$ ratios. For example, an enzyme *E* that catalyses three reactions (*R1*, *R2* and *R3*) is transformed into *E* _1 (highest $k_{cat}$, lowest MW/ $k_{cat}$ ratio) catalysing *R1*; *E* _2 (second highest $k_{cat}$, second lowest MW/ $k_{cat}$ ratio) catalysing *R2*, and *E* _3 (lowest $k_{cat}$, highest MW/ $k_{cat}$ ratio) catalysing *R3*. These pseudoenzymes comprises a subpool that draw resources from the enzyme pool, which corresponds to the individual enzyme pool as represented in GECKO 3 and is specific to one enzyme. Each enzyme pool then draws resources from the total protein pool, which is obtained from an exchange reaction as originally represented in GECKO 3. Lastly, GECKO 3 introduces a new structure in the YAML and MAT file formats, the “.ec” structure, where all enzyme information is stored. Given that CORAL changes how enzymes are integrated in the model, most of this information no longer matches what is present in the model after the changes take place. The third step updates all enzyme information and makes it available at the new “.und” structure, while the original “.ec” structure remains available.

#### Flux variability analysis

To evaluate the ranges of metabolic fluxes and of enzyme usages in the CORAL model, we performed flux variability analysis (FVA). We compared two scenarios: one where the underground metabolism reactions are present, and another where these reactions are blocked (upper and lower bounds set to zero). To perform this analysis, we first maximized and minimized the flux v of reaction j to find its flux range:

(P1) $$\max_{v}/\underset{v}{\min\;}v_{j}$$

subject to:

(Equation 1) S·v=0

(Equation 2) $$v_{\min}\leq v\leq v_{\max}$$

(Equation 3) $$v_{j}\leq k_{cat}^{i,j}\cdot E_{s,i}$$

(Equation 4) $$k_{cat}^{l,j}\cdot E_{s,l}\leq k_{cat}^{i,j}{\cdot E}_{s,i}$$

(Equation 5) $$\sum E_{s,i}=E_{s}$$

(Equation 6) $$\sum E_{s}=E_{tot}$$

where S is the stoichiometric matrix, v is the flux distribution vector, $v_{\min}$ is the flux lower bound vector, $v_{\max}$ is the flux upper bound vector, $k_{cat}^{i,j}$ is the $k_{cat}$ value for the enzyme subpool i, catalysing the reaction j, E is the enzyme usage in the enzyme s, subpool i, $E_{s,l}$ is the enzyme usage for any subpool m that is not catalysing the main reaction, $k_{cat}^{l,j}$ is the $k_{cat}$ value for enzyme l, and $E_{tot}$ is the total protein pool. In Equations 4, 11, and 18, we introduce a physiologically relevant constraint whereby more efficient enzymes (i.e., higher $k_{cat}$ values) would require allocation of enzyme resources that allows at least as large flux than the side reactions. In other words, we aim to model the prioritization of enzyme resources based on catalytic efficiency of the enzyme. We note that this inequality is implemented for the succession of the ordered $k_{cat}$ values associated with the functions of a promiscuous protein. Next, we performed FVA on the enzyme usage pseudoreactions using the same constraints as before, but with the following objective function:

(P2) $$\max_{E}/\min_{E}\;E_{s,i}$$

We were also interested in whether differences in culture conditions could impact the flux variability. Thus, in addition to the batch culture simulations above, we simulated glucose-limited chemostats by fixing the growth rate to the dilution rate, adding the following constraint to the optimization problems above:

(Equation 7) $$v_{bio}=\mu$$

where $v_{bio}$ is the flux through the biomass pseudoreaction, and μ is the specific growth rate.

#### Simulation of metabolic defects

Given that most resources are allocated to the main reaction,^17^ and given the fact that promiscuous reactions can compensate metabolic defects,^4^ we investigated how the fluxes in the metabolic network redistribute to accommodate this defect. To this end, we simulated how the enzyme resources are distributed from the main reaction to side reactions when the main reaction is blocked. We first used a modified implementation of parsimonious FBA (pFBA), whereby we minimize the sum of enzyme subpools instead of sum of fluxes. Here we obtain an initial, reference distribution of enzyme usage we consider as the wild-type (WT) distribution:

(P3) $$\underset{E}{\min\;E^{subpools}}=\sum_{s=1}^{n}E_{s}$$

subject to:

(Equation 8) S·v=0

(Equation 9) $$v_{\min}\leq v\leq v_{\max}$$

(Equation 10) $$v_{j}\leq k_{cat}^{i,j}\cdot E_{s,i}$$

(Equation 11) $$k_{cat}^{l,j}\cdot E_{s,l}\leq k_{cat}^{i,j}{\cdot E}_{s,i}$$

(Equation 12) $$\sum E_{s,i}=E_{s}$$

(Equation 13) $$\sum E_{s}=E_{tot}$$

(Equation 14) $$v_{bio}=\mu$$

where n is the number of enzymes.

After finding the initial, reference enzyme subpool distribution, we used the predicted enzyme subpool usages values to constrain the model (allowing 5% flexibility) in a second round of simulation, where we block the main reaction catalysed by an enzyme $E_{s}$. We implement this reaction blockage by setting the enzyme usage value of the subpool $E_{s,1}$, responsible for the main reactions, to zero, indicating that no enzyme resources will be available for that reaction. Considering Equations 3, 10, and 17, this also means that the flux for that reaction will also be zero. This is done for each enzyme s one by one, while we optimize growth:

(P4) $$\underset{v}{\max\;}v_{bio}$$

subject to:

(Equation 15) S·v=0

(Equation 16) $$v_{\min}\leq v\leq v_{\max}$$

(Equation 17) $$v_{j}\leq k_{cat}^{i,j}\cdot E_{s,i}$$

(Equation 18) $$k_{cat}^{l,j}\cdot E_{s,l}\leq k_{cat}^{i,j}{\cdot E}_{s,i}$$

(Equation 19) $$\sum E_{s,i}=E_{s}$$

(Equation 20) $$\sum E_{s}=E_{tot}$$

(Equation 21) $$E_{s}^{subpools}\cdot 0.95\leq E_{s}\leq E_{s}^{subpools}\cdot 1.05$$

(Equation 22) $$E_{s,1}=0$$

where $E_{s}^{subpools}$ is the enzyme subpool usage for subpool s as predicted in the previous step.

Next, we investigated how these metabolic defects can impact growth. To this end, we performed a pairwise block of the main reactions and optimized for growth. For all pairs of enzyme subpools in main reactions in the model, we set their usage values to zero:

(P5) $$\underset{v}{\max\;}v_{bio}$$

subject to:

(Equation 23) S·v=0

(Equation 24) $$v_{\min}\leq v\leq v_{\max}$$

(Equation 25) $$v_{j}\leq k_{cat}^{i,j}\cdot E_{s,i}$$

(Equation 26) $$k_{cat}^{l,j}\cdot E_{s,l}\leq k_{cat}^{i,j}{\cdot E}_{s,i}$$

(Equation 27) $$\sum E_{s,i}=E_{s}$$

(Equation 28) $$\sum E_{s}=E_{tot}$$

(Equation 29) $$E_{s,1}=0$$

(Equation 30) $$E_{d,1}=0$$

where the subpool $E_{d,1}$ is the subpool 1 from any enzyme d that is not the same as the enzyme s. To further assess the effect of these metabolic defects on growth, we performed double knockouts in the conventional GEM, iML1515u, deleting pairs of genes following classical GPR Boolean implementations and optimizing growth as in P5, excluding the enzyme constraints.

Lastly, we were also interested in how blocking other subpools, besides $E_{s,1}$ and $E_{d,1}$, affect growth. We blocked pairs of subpools from $E_{s,2}$, $E_{d,2}$ to $E_{s,5}$, $E_{d,5}$, since there are few enzymes with more than five subpools.

#### Flux-sum analysis

To further assess the capabilities of the CORAL model, we investigated the metabolite turnover (flux-sum analysis^18^) across the optimization problems we solved previously. This ensured that not only we take an enzyme- and reaction-centric overview, but also provide a metabolite-centric assessment. To calculate flux-sums, we apply the following equation:

(Equation 31) $$\phi_{i}=0.5\;\sum_{j}\left|S_{i,j}v_{j}\right|$$

where $\phi_{i}$ is the flux-sum of metabolite i, $S_{i,j}$ is the stoichiometric coefficient of metabolite i participating in reaction j, and $v_{j}$ is the flux through reaction j.

### Quantification and statistical analysis

To check for duplicated or highly similar reactions during model curation, we calculated the cosine similarity and defined a threshold of 90% for similarity. We calculated the ratio of how much resource each enzyme subpool draws from the enzyme pool by dividing the subpool resource usage by the total pool usage ($E_{s,j}/E_{s}$), as shown in the results section. We calculated the difference in flux-sums between the wildtype and the defective network solutions by subtracting the values from the defective solution with the values from the wildtype ($\phi_{i}^{del}-\phi_{i}^{WT}$), as shown in Figure 3 legend. For distribution of growth rate ratios, we calculated the ratios by dividing the predicted growth rates of the wildtype and defective strain (μWTμdel), as shown in Figure 4 legend. All quantifications, calculations and analyses were performed in MATLAB (The MathWorks Inc., Natick, Massachusetts).

## Supplemental information

Document S1. Figures S1–S4

Table S1. Excel file containing the list of added reactions and their corresponding *k*_*cat*_ values, predicted from either DLKcat or TurNuP

Table S2. Excel file containing the list of subpools and their corresponding usage values of the enzyme pool

Table S3. Excel file containing the list of subpool pairs for blocked pairs of main reactions and their corresponding growth rate before and after being blocked, related to Figure 4

Table S4. Excel file containing the list of metabolites evaluated during flux-sum analysis after blocking a single main reaction for both DLKcat- and TurNuP- parameterized models, related to Figure 3
