# Supplementary material for: Protein-constrained models pinpoints the role of underground metabolism in robustness of metabolic phenotypes
Source: iScience. 2025 Feb 28;28(3):112126. doi: 10.1016/j.isci.2025.112126 (PMC11951047; doi:10.1016/j.isci.2025.112126)
Supplement: Document S1. Figures S1–S4 [file mmc1.pdf]

iScience, Volume *28*

Supplemental information

Protein-constrained models pinpoints the role of underground metabolism in robustness of metabolic phenotypes

Maurício Alexander de Moura Ferreira, Eduardo Luís Menezes de Almeida, Wendel Batista da Silveira, and Zoran Nikoloski

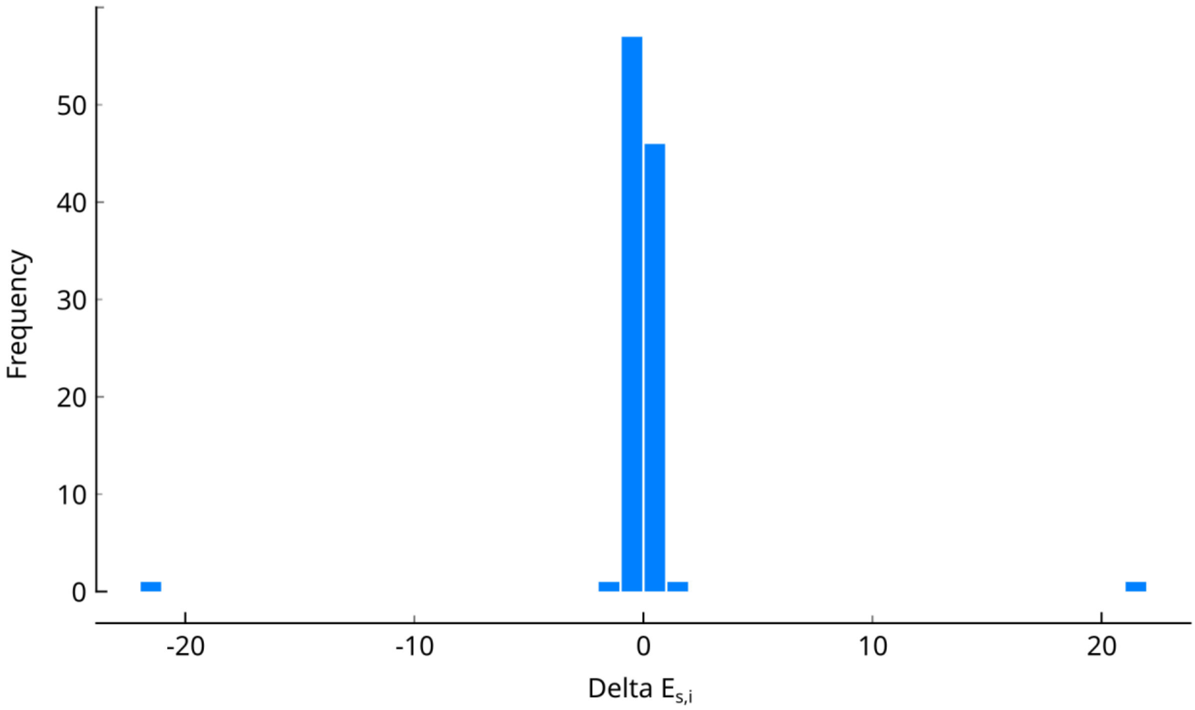

Figure S1. **Changes in enzyme subpool usage after blocking a single main reaction.** We calculated delta by subtracting the enzyme subpool usage of the wildtype from the enzyme subpool usage of the defective network solutions.

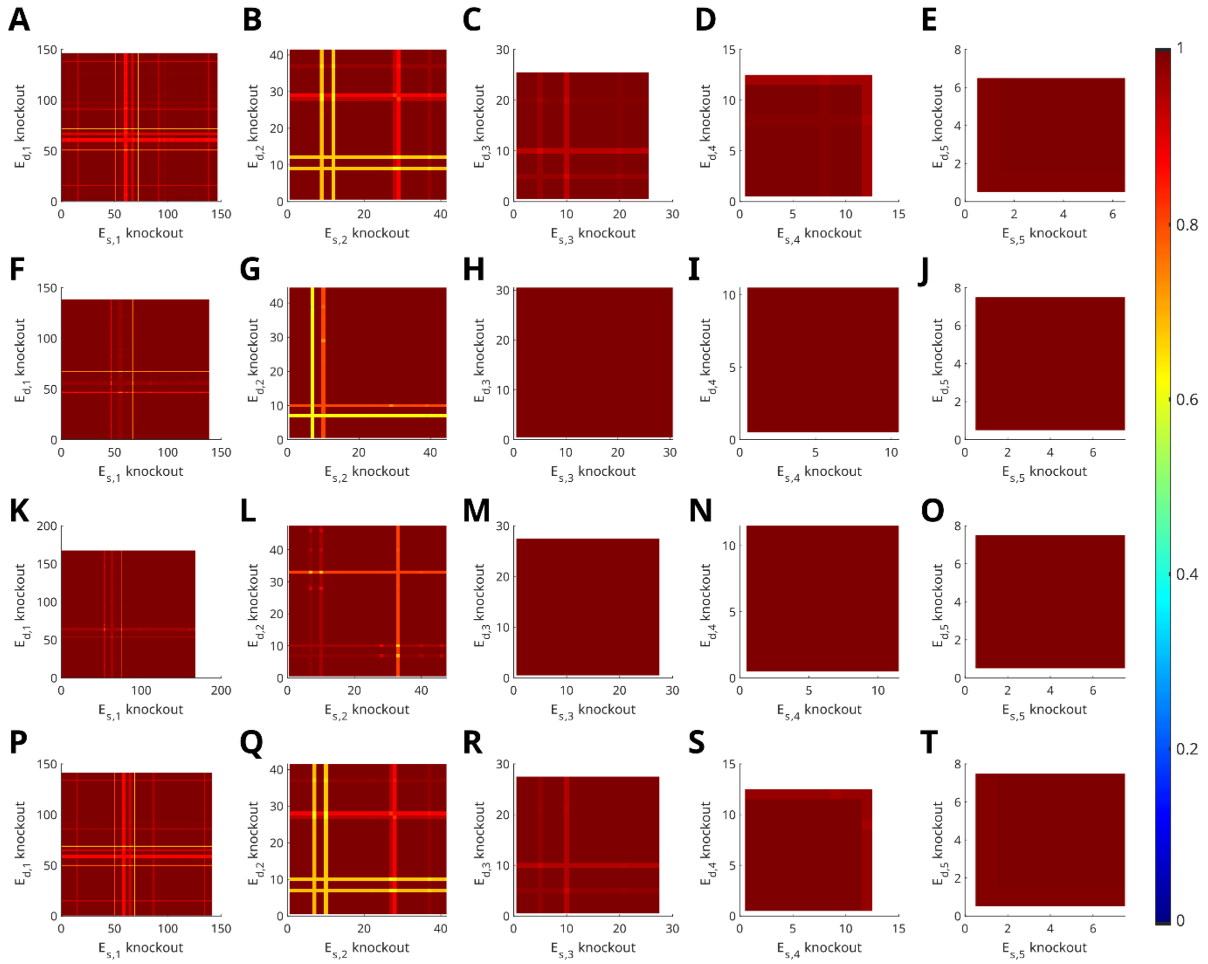

Figure S2. **Impact on growth after double metabolic defects considering alternative carbon sources. A)** Impact on growth after blocking a pair of main reactions (first subpool, $E_{s,1}$) using arabinose as carbon source. **B)** Second subpool ($E_{s,2}$). **C)** Third subpool ($E_{s,3}$). **D)** Fourth subpool ($E_{s,4}$). **E)** Fifth subpool ($E_{s,5}$). **F)** Impact on growth after blocking a pair of main reactions (first subpool, $E_{s,1}$) using fructose as carbon source. **G)** Second subpool ($E_{s,2}$). **H)** Third subpool ($E_{s,3}$). **I)** Fourth subpool ($E_{s,4}$). **J)** Fifth subpool ($E_{s,5}$). **K)** Impact on growth after blocking a pair of main reactions (first subpool, $E_{s,1}$) using fucose as carbon source. **L)** Second subpool ($E_{s,2}$). **M)** Third subpool ($E_{s,3}$). **N)** Fourth subpool ($E_{s,4}$). **O)** Fifth subpool ($E_{s,5}$). **P)** Impact on growth after blocking a pair of main reactions (first subpool, $E_{s,1}$) using xylose as carbon source. **Q)** Second subpool ($E_{s,2}$). **R)** Third subpool ($E_{s,3}$). **S)** Fourth subpool ($E_{s,4}$). **T)** Fifth subpool ($E_{s,5}$). Related to Figure 5.

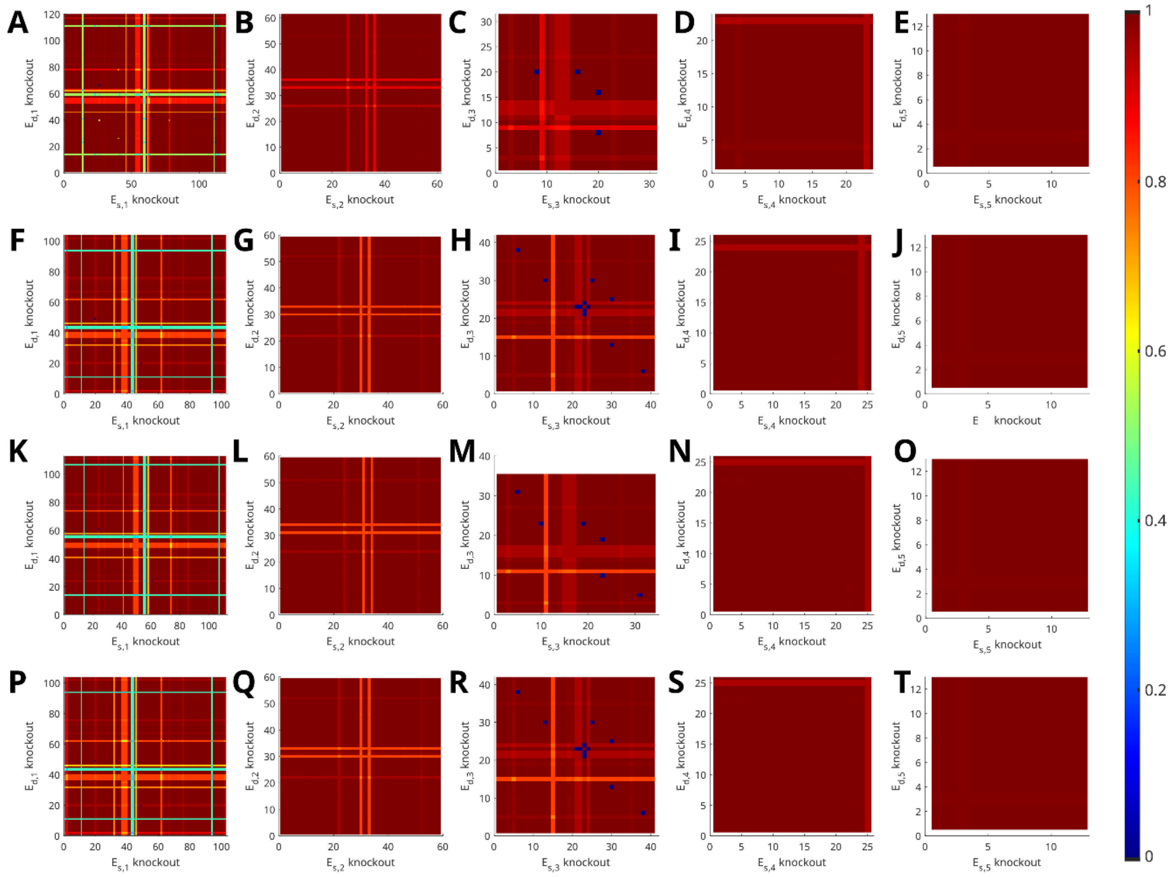

Figure S3. **Impact on growth after double metabolic defects considering organic acids as carbon sources instead of sugars. A)** Impact on growth after blocking a pair of main reactions (first subpool, $E_{s,1}$) using acetate as carbon source. **B)** Second subpool ($E_{s,2}$). **C)** Third subpool ($E_{s,3}$). **D)** Fourth subpool ($E_{s,4}$). **E)** Fifth subpool ($E_{s,5}$). **F)** Impact on growth after blocking a pair of main reactions (first subpool, $E_{s,1}$) using citrate as carbon source. **G)** Second subpool ($E_{s,2}$). **H)** Third subpool ($E_{s,3}$). **I)** Fourth subpool ($E_{s,4}$). **J)** Fifth subpool ($E_{s,5}$). **K)** Impact on growth after blocking a pair of main reactions (first subpool, $E_{s,1}$) using glutamate as carbon source. **L)** Second subpool ($E_{s,2}$). **M)** Third subpool ($E_{s,3}$). **N)** Fourth subpool ($E_{s,4}$). **O)** Fifth subpool ($E_{s,5}$). **P)** Impact on growth after blocking a pair of main reactions (first subpool, $E_{s,1}$) using glutamine as carbon source. **Q)** Second subpool ($E_{s,2}$). **R)** Third subpool ($E_{s,3}$). **S)** Fourth subpool ($E_{s,4}$). **T)** Fifth subpool ($E_{s,5}$). Related to Figure 5.

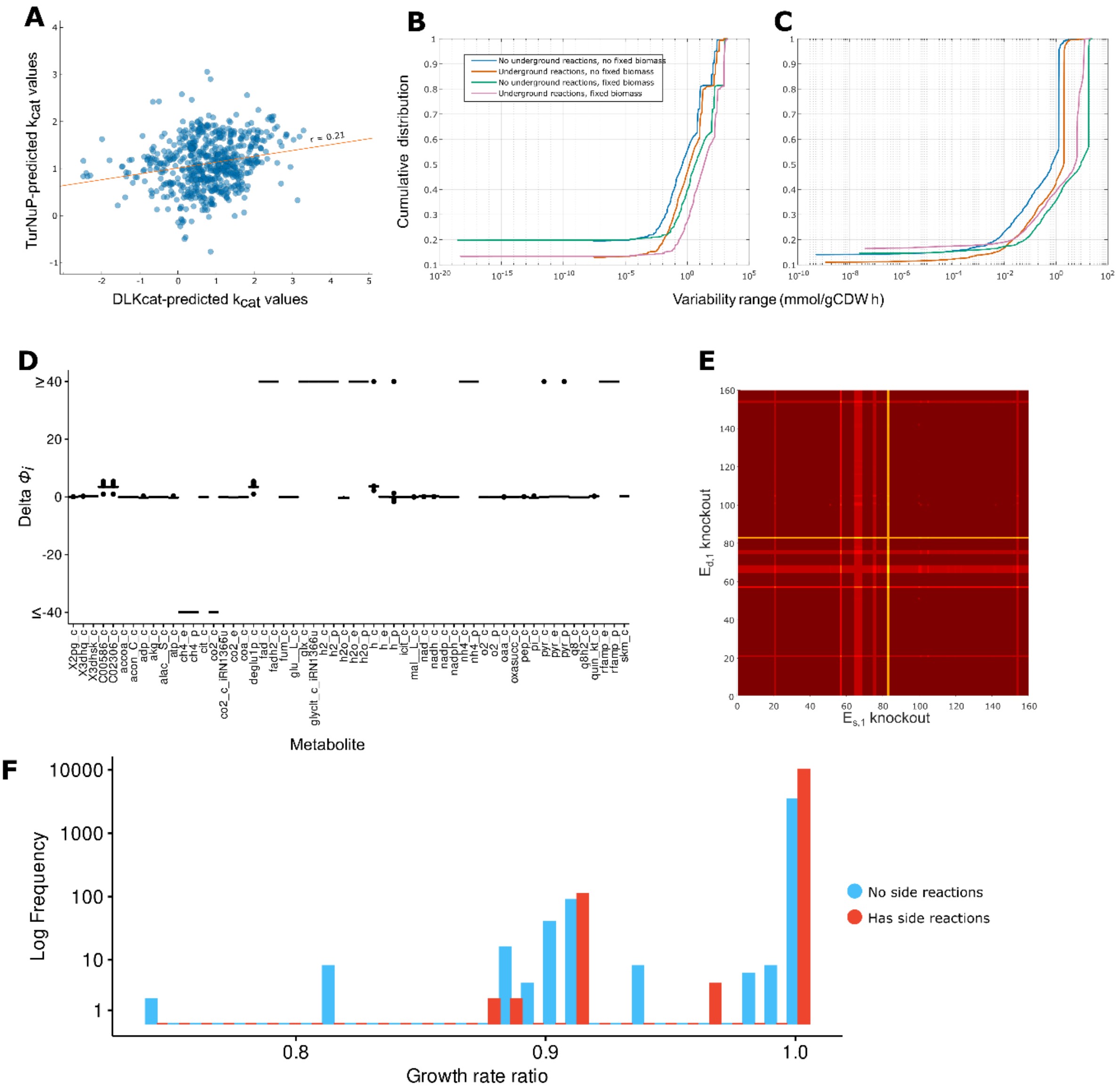

Figure S4. **Robustness analysis of CORAL using TurNuP-predicted k_{cat} values.** We performed all analyses as done when using DLKcat-predicted k_{cat} values. **A)** Comparison of DLKcat- and TurNuP-predicted k_{cat} values. **B)** Flux variability for metabolic reactions only (excluding all GECKO- and CORAL-related pseudoreactions). **C)** Flux variability for enzyme subpool usage. D) Difference in flux-sums between the wildtype and the defective network solutions. Full names of the metabolites appearing on the x-axis are provided in Table S4. **E)** Impact on growth after blocking a pair of main reactions. **F)** Growth rate ratios obtained from main reaction pairs that did not affect growth or affected it by less than 1%. **G)** Growth rate ratios obtained from main reaction pairs that affected growth by at least 1%. Related to Figures 2 to 5.
